# The Effects of Anterior Cruciate Ligament Reconstruction on Individual Quadriceps Muscle Thickness and Circulating Biomarkers

**DOI:** 10.3390/ijerph16244895

**Published:** 2019-12-04

**Authors:** Jae-Ho Yang, Seung-Pyo Eun, Dong-Ho Park, Hyo-Bum Kwak, Eunwook Chang

**Affiliations:** 1 Department of Kinesiology, Inha University, Incheon 22201, Korea; wdsa122@gmail.com (J.-H.Y.); dparkosu@inha.ac.kr (D.-H.P.); kwakhb@inha.ac.kr (H.-B.K.); 2 KOSMED Orthopedic Clinic, Seoul 06014, Korea; drkosmed@gmail.com

**Keywords:** anterior cruciate ligament reconstruction, quadriceps, vastus intermedius, muscle atrophy, myostatin

## Abstract

Anterior cruciate ligament reconstruction (ACLR) frequently results in quadriceps atrophy. The present study investigated the effect of ACLR on the muscle thickness of the different constituent muscles of the quadriceps and circulating biomarkers related to muscle atrophy and hypertrophy. Fourteen subjects underwent anterior cruciate ligament reconstruction following injury. Quadriceps muscle thicknesses were measured using ultrasound, and circulating biomarkers in the blood were measured using enzyme-linked immunosorbent assays (ELISAs) at the preoperative visit (PRE) and at two postoperative visits (PO1, PO2) in the early stages post-surgery. Differences between time points were analyzed using one-way repeated measures analysis of variance (ANOVA) tests. The most important finding was that severe muscle atrophy occurred in the vastus intermedius (VI) after ACLR (PRE: 20.45 ± 6.82 mm, PO1: 16.05 ± 6.13 mm, PO2: 13.18 ± 4.7 mm, F = 59.0, *p* < 0.001). Furthermore, the myostatin level was slightly increased, and IGF-1 was significantly reduced throughout the entire period. Therefore, we suggest that inducing selective hypertrophy in the vastus intermedius during the process of rehabilitation would be important for athletes and individuals who engage in explosive sports. Moreover, inhibiting myostatin level increases and maintaining IGF-1 levels in the early phase of recovery after ACLR to prevent muscle atrophy may provide a pharmaceutical option for rehabilitation after anterior cruciate ligament injury.

## 1. Introduction

Anterior cruciate ligament (ACL) injury is common in individuals who participate in explosive sports such as soccer, basketball, and American football. ACL tears account for over 60% of knee injuries in rapid movement-related sports [1]. Most ACL injuries require anterior cruciate ligament reconstruction (ACLR) to restore knee joint stabilization. However, patients with ACLR have shown persistent atrophy and weakness of their quadriceps [2] after completion of the postoperative rehabilitation program. Previous studies have reported that muscle atrophy following ACLR persisted between six and eighteen months [3,4], potentially resulting in functional deficits. In addition, previous studies have demonstrated that quadriceps torque deficits can exceed 30% at six months following ACLR—a time when many athletes are cleared to return to action [5,6]. Therefore, it could be essential to restore quadriceps muscle thickness to allow optimal knee joint function.

The quadriceps femoris, a large muscle located in the anterior compartment of the thigh, consists of the rectus femoris (RF), vastus intermedius (VI), vastus lateralis (VL), and vastus medialis (VM). The optimal function of the quadriceps muscle is important for athletic movement. Specifically, previous studies showed that greater knee extension peak torque was associated with greater vertical jump height [7,8] and faster sprint performances [7,9]. In addition, the quadriceps muscle plays a significant role in providing knee joint stabilization and injury prevention [10]. According to previous investigations, while the primary function of the quadriceps muscle is knee joint extension, each quadriceps component performs specific roles in optimal knee function [11,12,13]. For example, the vastus medialis oblique (VMO) plays an important role in medial patella stabilization against the VL [12]. Moreover, the VI is not only a primary knee extensor but also the highest antagonistic coactivator during isometric knee flexion [11,13]. Although each quadriceps component functions differently, there is limited evidence regarding the atrophy of specific quadriceps components following ACLR. Understanding the atrophy of individual quadriceps components would be helpful for health care professionals when treating ACL patients.

Although surgical reconstruction following ACL injuries is helpful in restoring some joint kinematics and proprioception, many patients still develop quadriceps atrophy and weakness [6]. Several researchers have shown that quadriceps atrophy and weakness following ACLR are induced from neuromuscular impairment, such as arthrogenic muscle inhibition (AMI), proprioception deficits, and a reduction in postural control [14,15,16]. Based on this research, present rehabilitation strategies usually focus on strength and neuromuscular training using cryotherapy, electrostimulation, and electromyographic feedback [17,18]. However, because the morphological and functional features of the muscle are changed by atrophy or hypertrophy-inducing signaling molecules, providing better treatment to ACLR patients may require developing new therapeutic interventions based on the release of signaling molecules after ACLR to prevent quadriceps atrophy and weakness.

Several cytokines and signaling molecules are known to promote muscle hypertrophy or atrophy through various mechanisms. However, little is known about the change in the levels of these biomarkers following ACLR. Myostatin, as a member of the transforming growth factor-β (TGF-β) family, not only inhibits muscle growth and development by preventing unopposed activin-induced muscle hypertrophy but also induces muscle atrophy by activating the ubiquitin-proteasome pathway [19]. TGF-β also induces muscle atrophy and weakness via the ubiquitin-proteasome pathway [20]. Tumor necrosis factor-α (TNF-α) is a central regulator of inflammation, involved in the acute phase of the inflammatory response [21]. On the other hand, insulin-like growth factor-1 (IGF-1) is the most well-known hypertrophy-related biomarker and activates Akt/mTOR signaling [22]. Decorin is also a well-studied hypertrophic factor that increases hypertrophy by TGF-β in the extracellular matrix (ECM) [23], and adiponectin possesses anti-inflammatory effects through various mechanisms [24]. However, the change in the levels of circulating biomarkers following ACLR is not yet clearly understood. Therefore, the purpose of this study was to compare thickness changes in the individual components of the quadriceps muscle and examine the change in circulating biomarker levels before and after ACLR. 

## 2. Materials and Methods

### 2.1. Patients

This study was approved by Inha University’s Institutional Review Board. An a priori power analysis was performed to calculate the number of subjects required; a minimum sample size of 15 subjects was required to detect a medium to large effect size at a β-power of over 0.8. Twenty subjects were recruited after factoring for potential subject dropouts. Twenty participants who had unilateral ACL injuries and were scheduled for reconstructive surgery were recruited in this study after meeting the orthopedic surgeon prior to surgery. Patients who had previous injuries to the involved knee or had a history of myopathy or rheumatological diseases were excluded from participation in the study. Before measurement began, participants were asked to read and agree to the participation agreement. Finally, 14 recreationally active patients (10 men and 4 women) completed the study. The mean age of the patients was 30.4 ± 5.9 years (range: 22–42 years), mean height was 170.8 ± 8.6 cm (range: 151–184 cm), mean body mass was 69.9 ± 10.8 kg (range: 48–85 kg), and body mass index was 23.8 ± 2.3 kg/m^2^ (range: 20.9–28.7 kg/m^2^) (Table 1).

### 2.2. Study Design

**Figure 1:**
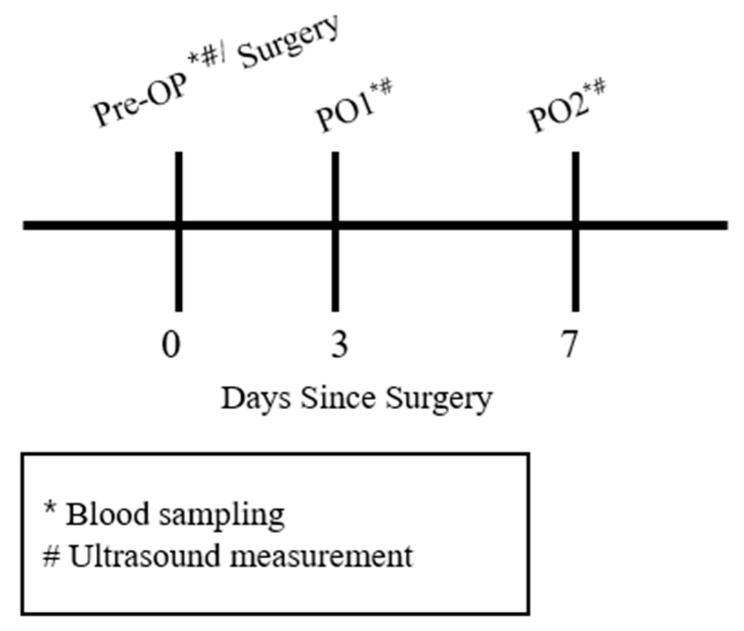
Overview of the study design demonstrating the preoperative (PRE) surgery and postoperative (PO) study time points.

An overview of this study design is demonstrated in Figure 1. Before surgery, patients filled out a participation agreement. Next, the researcher conducted an ultrasound measurement, and blood sampling was performed by a nurse who worked in the clinic. The first (PO1) and second (PO2) postoperative visits for blood sampling and quadriceps ultrasound measurements occurred three and seven days after surgery, respectively.

### 2.3. Experimental Protocol

#### 2.3.1. Blood Sampling

Venous blood was drawn from the patients’ median cubital vein or cephalic vein into a serum separator tube containing clot activator and serum separator gel 1 h before surgery and at PO1 and PO2. The collected blood was kept in the serum separator tube for 30 min for solidification and was centrifuged at 4000 RPM for 15 min. The serum was then immediately collected from the tube and placed in a 1.5 mL tube and stored at −80 °C for blood analysis. 

#### 2.3.2. Muscle Thickness Measurement

**Figure 2:**
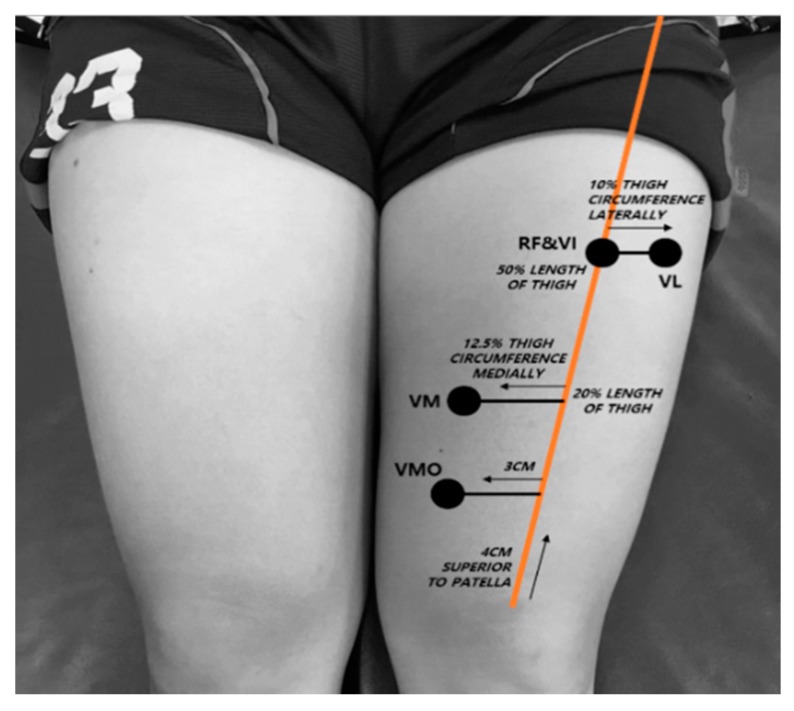
Quadriceps muscle thickness measurement site.

**Figure 3:**
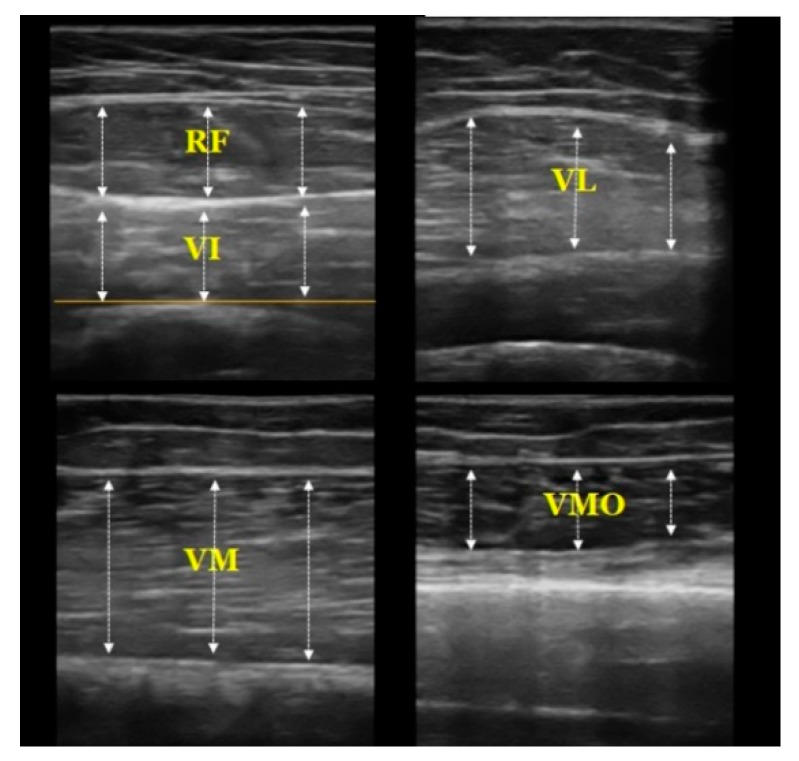
The images used to measure the muscle thickness of the components of the quadriceps.

Quadriceps muscle thickness was measured in a random order. Quadriceps muscle thickness was imaged using a portable ultrasound (7.5 MHz transducer, Healcerion, Seoul, Korea). The RF and VI were measured at halfway along the line between the anterior superior iliac spine (ASIS) and superior patella pole, and VL was measured at 10% of the thigh circumference laterally from the RF–VI measurement site. The VM was measured at 12.5% of the thigh circumference medially from the line between the ASIS and superior patella pole [25]. The VMO was measured 4 cm superior and 3 cm medial from the superior patella pole [26], as seen in Figure 2. Because the RF, VI, and VL muscles have a concave or convex shape, we used an average value of three lines placed at regular intervals along each muscle in the recorded images. The VM and VMO were measured in the longitudinal plane because of their irregular shape, and the other muscles were measured in the transverse plane. The muscle thickness of the RF was defined as the distance between the superficial border of the muscle and the deep border of the muscle. The thickness of the VI was defined as the distance between the superficial border of the muscle and the line of the superficial border of the femur. The muscle thicknesses of the VL, VM, and VMO were defined as the distance between the superficial border of the muscle and the inferior border of the muscle [25], as shown in Figure 3. The images were recorded when the femur was visible in the center of the screen and the boundaries of the muscles were clearly visible. The three valid images were recorded in a random order for each muscle. ImageJ software (National Institutes of Health, Bethesda, MD) was used to measure the muscle thickness. Muscle thickness was defined as the average value of three lines placed at equal intervals in the muscle belly, as depicted in Figure 3. The average value of the three images was utilized in statistical analyses. 

### 2.4. Measurement of Biomarkers

The concentration of circulating biomarkers was analyzed using serum samples in duplicate for each time point. Each biomarker analysis was conducted according to the manufacturer’s instructions. An enzyme-linked immunosorbent assay (ELISA) was performed to measure the circulating levels of myostatin (R&D Systems, Minneapolis, MN, USA), TGF-β (R&D Systems), TNF-α (Cohesion Bioscience, London, UK), IGF-1 (R&D Systems), adiponectin (Phoenix, NY, USA), and decorin (Boster Bio, Pleasanton, CA, USA).

### 2.5. Statistical Analysis

Data are presented as the mean ± standard error of the mean (SEM). Jamovi 1.0.7.0 software (Jamovi Project, 2018) was used for identifying significant differences in muscle thickness and circulating biomarker levels between time points using one-way repeated measures analysis of variance (ANOVA) (α = 05). Tukey’s post-hoc test was used to identify differences between each time point. We also calculated Cohen’s d effect sizes to evaluate the practical significance of the results.

## 3. Results

### 3.1. Effects of Anterior Cruciate Ligament Reconstruction on Individual Quadriceps Muscle Thickness

**Figure 4:**
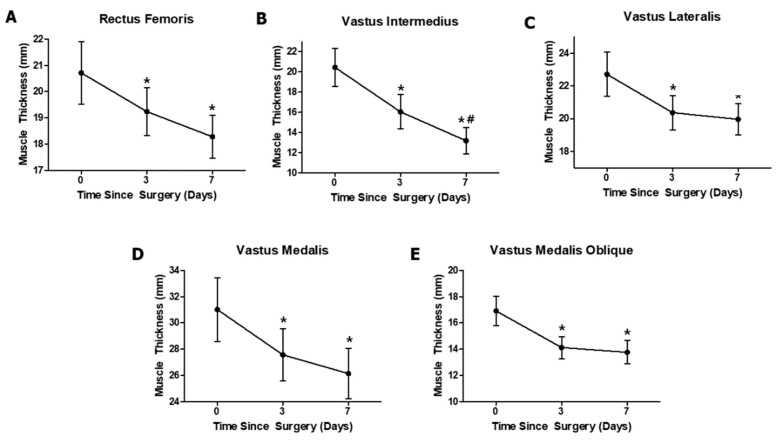
Changes in muscle thickness over the entire period of study. (**A**) Rectus femoris (RF), (**B**) vastus intermedius (VI), (**C**) vastus lateralis (VL), (**D**) vastus medialis (VM), and (**E**) vastus medialis oblique (VMO). Values are expressed as the mean ± standard error. * *p* < 0.05 compared with the preoperative (PRE: Days 0) values. # *p* < 0.05 compared with the first postoperative visit (PO1: Days 3).

The RF, VI, VM, and VMO muscles showed a significant reduction in thickness, and post-hoc testing revealed that the PRE thickness was greater than those at PO1 and PO2 (Table 2 and Figure 4). Importantly, the VI muscle only showed a reduction in thickness between PO1 and PO2 (Table 2 and Figure 4B). The VI alone did not exhibit any significant reduction in muscle thickness across the three time points. 

### 3.2. Effect of Anterior Cruciate Ligament Reconstruction on Circulating Biomarkers

**Figure 5:**
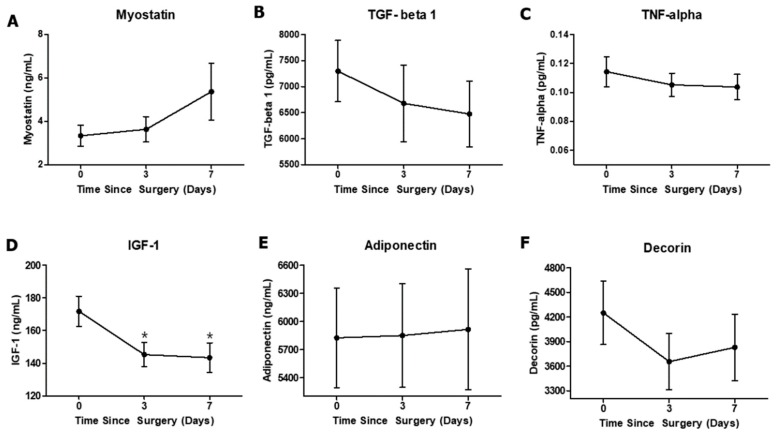
Changes in serum levels of circulating biomarkers over the entire period of the study. (**A**) Myostatin, (**B**) TGF-beta 1, (**C**) TNF-alpha, (**D**) IGF-1, (**E**) adiponectin, and (**F**) decorin. Values are expressed as mean ± standard error. * *p* < 0.05 compared with PRE.

Although there were no significant differences between the three time points for the other biomarkers, the myostatin level showed a medium effect size between PRE and PO2 (d = 0.55) (Table 3 and Figure 5A). The IGF-1 level was significantly decreased at PO1 and PO2 compared with that at PRE, and there were large effect sizes between PRE and PO1 (d = 0.84) and between PRE and PO2 (d = 0.82) (Table 3 and Figure 5D). TGF-Beta 1, TNF-alpha, adiponectin, and decorin did not exhibit any significant differences between the three time points (Figure 5B,C,E,F). 

## 4. Discussion

The purpose of this study was to determine the effect of ACLR on individual quadriceps muscle thickness and circulating biomarkers. Previous studies have reported that many patients suffered quadriceps atrophy for several months to years after ACLR [2,27]. However, it is unclear how individual components of the quadriceps decreased in size a week after ACLR. Because each quadriceps component has a different function [11,12,28], it is important for health care professionals to identify the atrophy patterns of the quadriceps. Thus, in this study, we measured individual quadriceps muscle thicknesses using ultrasound to identify the quadriceps atrophy pattern in the early phase after ACLR. All quadriceps muscle thicknesses were significantly decreased at PO1 compared with that at PRE, and the VI was the only muscle that showed a significant decrease in thickness at PO2 compared with that at PO1. In the case of biomarkers, our result shows that only the IGF-1 level was significantly reduced at PO1 and PO2 compared with that at PRE after ACLR. However, the change in myostatin level between PRE and PO1 had a medium effect size.

According to previous studies, the VI is the most important knee extensor in the quadriceps and is related to the rate of torque development in its early stages, suggesting that the VI is crucial for athletes who need explosive movements [11]. Given these previous studies, the VI muscle may have properties that are difficult to use in daily life and the early phases of rehabilitation. Thus, it is essential for athletes or individuals who participate in explosive sports with an ACL injury to recover the function and size of the VI muscle by utilizing effective exercises to selectively stimulate the VI muscle [2,18]. Importantly, the mechanism of continuous VI atrophy after ACLR is unclear; thus, it could be necessary to investigate the types of rehabilitation exercise to prevent VI atrophy in the future. 

Many studies have reported that decreased neuromuscular control and proprioception deficit after ACLR were the primary reasons for quadriceps muscle atrophy [14,16]. However, from a physiological perspective, the change in cytokine levels after ACLR could directly or indirectly influence muscle size. Myostatin is the representative atrophy-inducing cytokine. Several studies have reported that mutations in the myostatin gene induce tremendous hypertrophy [19,27]. However, in physiologically normal conditions, myostatin induces muscle atrophy via the Fox O pathway and interacts with the activin receptor complex to prevent unopposed activin-induced muscle hypertrophy via a signaling cascade [19,29]. One study reported that ACL tears promote skeletal muscle myostatin expression and fibrogenic cell expansion and decrease muscle quality [29]; another study reported that myostatin levels tend to increase up to two weeks after ACL surgery [30]. Our study supports the above studies in that the change in myostatin levels exhibited a small effect size at PO1 (three days after ACLR) and a medium effect size at PO2 (seven days after ACLR) compared with that at PRE. Thus, inhibition of the myostatin level would be an important pharmacological option to restore patients to their pre-injury physical condition and accelerate a return to sports. 

Other muscle atrophy-related factors, TGF-β and TNF-α, exhibited decreases with small effect sizes at PO1 and PO2 compared with the levels at PRE. TGF-β is a cytokine closely related to myostatin, which directly induces muscle atrophy and severely reduces muscle force generation [20]. A previous study reported that the level of TGF-β decreased from before surgery to three days after surgery and increased significantly in the interval from three days after surgery to two weeks after surgery [30]; our study, however, showed only a tendency to decrease with a small effect size within a week. It can be expected that the levels of TGF-β tend to decrease immediately after surgery then increase gradually. The representative acute inflammatory biomarker, TNF-α, can directly induce muscle atrophy by activating the ubiquitin proteolytic system in muscle [22,31]. However, in this study, no change in the TNF-α level was detected. This result supports a previous study that found there was no difference in TNF-α level after ACLR [30]. In the case of hypertrophy-related biomarkers, IGF-1 is the only biomarker that showed a significant difference between the preoperative level and postoperative level. This result is in contrast with a previous study where the IGF-1 level did not significantly change throughout the course of the study [30]. A potential explanation for why only IGF-1 showed a significant difference is because IGF-1 is the representative hypertrophy-related cytokine but also contributes to muscle regeneration and the healing process of connective tissue [31,32,33,34]. This result suggests that maintaining the IGF-1 level may offer a way to safely restore muscle to its pre-injury condition after ACLR.

The mechanism of selective atrophy of the VI after ACLR has not been reported yet. Previous investigations showed quadriceps atrophy after knee placement and ACLR [35,36], but the specific muscle atrophy pattern was not well investigated. Investigating the mechanism of selective muscle atrophy is warranted, as it could be crucial for providing further treatment options such as pharmacological treatment. 

This study has several limitations. First, the rehabilitation of the subjects after surgery was not controlled. The frequency and intensity of rehabilitation can potentially affect the thickness of each muscle in the quadriceps and the biomarkers in the blood. Second, the inflammation-related biomarkers that could be produced at the initial injury were not controlled. Additionally, potential chronic metabolic disease in each patient was not controlled. Furthermore, the participants’ sex ratio (male: 10 and female: 4) was not manageable, as we were not able to match the sex of injured patients. In addition, the method of surgery may affect the variables, because subjects were operated on by one surgical specialist at the hospital. Finally, both male and female patients were included, therefore sex-related hormones were not controlled.

## 5. Conclusions

This study investigated the effect of ACLR on the muscle thickness of individual components of quadriceps and circulating biomarkers. The most important finding was that severe muscle atrophy occurred in the VI after ACLR. The levels of the hypertrophy-inducing biomarker, IGF-1, were significantly lower after ACLR. In addition, the levels of the atrophy-inducing biomarker, myostatin, did not significantly increase but showed clinical importance because of the medium effect size of the difference. 

## Figures and Tables

**Table 1:** Participant demographics.

Characteristic	
Age, year	30.4 ± 5.9
Height, cm	170.8 ± 8.0
Weight, kg	69.9 ± 10.8
Body mass index, kg/m^2^	23.8 ± 2.3
Days between injury and reconstruction, days	38.4 ± 76.7

**Table 2:** Mean ± standard deviation and percentage of quadriceps muscle thickness at each time points.

	PRE	PO1	PO2	F	p	η^2^p
RF (mm)	20.72 ± 4.28	19.24 ± 4.28 *	18.28 ± 18.28 *	7.30	0.009	0.36
VI (mm)	20.45 ± 6.82	16.05 ± 6.13 *	13.18 ± 4.7 *^,#^	59	<0.001	0.819
VL (mm)	22.7 ± 4.86	20.37 ± 3.8 *	19.97 ± 3.48 *	2.96	0.094	0.186
VM (mm)	31.02 ± 8.76	27.57 ± 7.24 *	26.13 ± 6.93 *	15.1	<0.001	0.537
VMO (mm)	16.91 ± 3.98	14.12 ± 3.01 *	13.77 ± 3.16 *	23.7	<0.001	0.646

* *p* < 0.05 compared with PRE. ^#^ *p* < 0.05 compared with PO1.

**Table 3:** Cohen’s d effect size of the circulating biomarkers between each timepoints.

	PRE vs. PO1	PRE vs. PO2	PO1 vs. PO2
**Myostatin**	0.15	0.55	0.46
**TGF-beta 1**	0.25	0.36	0.08
**TNF-alpha**	0.26	0.29	0.05
**Decorin**	0.43	0.28	0.12
**Adiponectin**	0.02	0.03	0.01
**IGF-1**	0.84	0.82	0.06

Effect size: 0.2 = small; 0.5 = medium; 0.8 = large.

## Abbreviations

RF	Rectus Femoris	VI	Vastus Intermedius
VL	Vastus Lateralis	VM	Vastus Medialis
VMO		Vastus Medialis Obliques	
